# In Vivo Optical Coherence Tomography Outcomes of Hypotony After Trabeculectomy Management with Autologous Blood Injection: A Single-Center Retrospective Study

**DOI:** 10.3390/jcm14093030

**Published:** 2025-04-27

**Authors:** Matteo Sacchi, Mattia Marchetti, Marta Pitzalis, Giacomo Tanda, Gianluca Monsellato, Gaia Li Calzi, Lorenza Ronchi, Stefano Dore, Paolo Nucci, Antonio Pinna

**Affiliations:** 1 Department of Medicine, Surgery and Pharmacy, University of Sassari, 07100 Sassari, Italy; stdore@uniss.it (S.D.); apinna@uniss.it (A.P.); 2 Ophthalmology Unit, Azienda Ospedaliero-Universitaria di Sassari, 07100 Sassari, Italy; dottormarchetti.mattia@gmail.com (M.M.); marta.pitzalis@outlook.it (M.P.); giacomotanda@hotmail.it (G.T.); loreronchi@icloud.com (L.R.); 3 Eye Clinic, San Giuseppe Hospital, IRCCS Multimedica, University of Milan, 20123 Milan, Italy; gianluca.monsellato@hotmail.com (G.M.); gaia.licalzi@unimi.it (G.L.C.); 4 Department of Biomedical, Surgical and Dental Sciences, University of Milan, 20122 Milan, Italy; paolo.nucci@unimi.it

**Keywords:** optical coherence tomography, blood injection, bleb, glaucoma, trabeculectomy, hypotony, hypotony maculopathy, over-filtering bleb

## Abstract

**Background**: This study aimed to report the efficacy and safety of peribleb autologous blood injections in patients with hypotony maculopathy following trabeculectomy. **Methods**: In this retrospective chart-review study, patients with hypotony maculopathy from over-filtering bleb following mitomycin C (MMC)-augmented trabeculectomy treated with ≥1 peribleb autologous blood injections, ≥12 months of follow-up, and macula optical coherence tomography (OCT) imaging were included. Patients with previous laser cyclophotocoagulation were excluded. Hypotony maculopathy was defined as choroidal folds in the macular region, as assessed by OCT. **Results**: Nine patients met the inclusion criteria (mean age 62.3 ± 17.0). The mean intraocular pressure (IOP) at hypotony maculopathy diagnosis was 3.8 ± 1.5 mmHg. Most (*n* = 7) patients received a single injection (4 injections *n* = 1, 5 injections *n* = 1). Significant improvements in mean overall IOP after blood injection were observed (8.3 ± 2.4 mmHg; *p* = 0.008). Improvements in visual acuity and the resolution of hypotony maculopathy were observed in patients requiring a single injection only. No intra-operative adverse events were recorded. Successful bleb surgical revision was performed for two patients refractory to blood injections. **Conclusions**: Peribleb autologous blood injection increased IOP, improved visual acuity, and resolved hypotony maculopathy in 5.3 weeks in 7/9 patients. This procedure is not a contra-indication for further surgical revision.

## 1. Introduction

Glaucoma is a neurodegenerative disease targeting the optic nerve and is caused by the apoptosis of retinal ganglion cells. Glaucoma is considered the leading cause of irreversible blindness worldwide, estimated to affect 110 million people by 2040 (3% of the global population) [1]. Ocular, systemic, and genetic risk factors are linked with glaucoma development. Among risk factors, the only modifiable variable is intraocular pressure (IOP) [2]. A timely reduction in IOP can prevent the development and progression of glaucoma.

The glaucoma treatment algorithm recommends surgery following (i) failed medication, (ii) failed laser approaches, (iii) patient allergy, or (iv) intolerance to topical drugs [2]. Filtering surgery aims to create a sclera fistula, enabling the outflow of aqueous humor from the anterior chamber to the subconjunctival space [3]. Surgical options for glaucoma filtering include trabeculectomy (with or without mitomycin C [MMC] augmentation), deep sclerectomy, minimally invasive bleb-forming glaucoma surgery (MIBS), and long-tube surgery. Evidence suggests that these surgical interventions are effective for IOP reduction but are not without complications [4,5,6,7]. Novel and minimally invasive surgical techniques have been growing in popularity; however, trabeculectomy is a commonly performed surgical intervention for glaucoma [8] and remains the gold-standard technique (i) for patients with angle-closure glaucoma and (ii) when disease control requires a low target IOP.

One of the most common post-surgical complications following trabeculectomy is hypotony. Hypotony may be associated with choroidal detachment, maculopathy, and/or optic nerve head edema [9,10,11]. Several strategies for the management of post-surgical hypotony have been reported, including autologous blood injection, ophthalmic viscoelastic devices (OVDs) [12,13], scleral lenses [14,15], sutures [16,17,18,19], corneal patch grafts [20], nylon sutures [21], and complete surgical revisions [22].

Autologous blood injection, due to its unique biological properties and clinical efficacy, has been employed since 1993. Autologous blood, rich in growth factors and cytokines, promotes healing and provides a scaffold for tissue regeneration [23] with a minimal risk of autoimmune or immunologic reactions [24]. Optical coherence tomography (OCT) provides high-resolution, cross-sectional images of the retina and other ocular structures, enabling in vivo, non-invasive assessment for diagnosing and monitoring hypotony maculopathy after filtration surgery [25,26]. Few case studies have reported outcomes of autologous blood injection in the treatment of post-trabeculectomy hypotony or provided evidence of immediate and mid-term morphological changes.

This study was designed to report the efficacy and safety of autologous blood injection into the peribleb in consecutive patients with post-trabeculectomy hypotony maculopathy from over-filtering bleb, assessed clinically and through in vivo OCT imaging of morphological changes. We also report clinical and morphological outcomes at 12 months.

## 2. Materials and Methods

We conducted a retrospective study of patients treated with trabeculectomy for uncontrolled IOP between March 2016 and June 2022 through a chart review of outpatient and surgical medical charts (electronic and paper based). The study was approved by the local ethics committee (Azienda Ospedaliera Universitaria Sassari) and was performed according to the Declaration of Helsinki. Patients were informed about the processing of personal data, and consent was obtained from all the patients before data were analyzed.

The criteria for study inclusion were adult patients (≥18 years) with evidence on OCT of hypotony maculopathy following trabeculectomy, over-filtering bleb, ≥1 autologous blood injection(s), follow-up of ≥12 months, and follow-up macula OCT scans of the choroidal folds. The criteria for study exclusion were bleb leakage; any treatments other than autologous blood injection for hypotony, with the exception of anterior chamber refill with OVD; and previous laser cyclophotocoagulation treatment. The minimum number of injections required for inclusion in the analysis was one. For patients who received more than one injection, there was no pre-defined minimum interval between injections.

Patients with uncontrolled IOP (measured by Goldmann applanation tonometry), ineffective topical therapy, clinically significant progression of glaucomatous damage (based on physician’s judgment), or intolerance to topical medication were treated with trabeculectomy. Visual-field (VF) (30-2 test, full-threshold, Humphrey field analyzer II 750; Carl Zeiss Meditec Inc., Dublin, CA, USA) damage progression was evaluated through trend analysis using the HFA Guided Progression Analysis software, version 2.0. OCT images were captured with a Spectralis instrument (Raster Scan mode; Heidelberg Engineering, Heidelberg, Germany) or the HD 5 Line Raster/HD Cross of the Zeiss Cirrus OCT (Carl Zeiss Meditec, Dublin, CA, USA). The same OCT platform was consistently used for each patient throughout the follow-up period

For the purpose of our analysis, the following demographic and clinical data were collected: age, gender, best-corrected visual acuity (BCVA), lens status, type of glaucoma, previous surgery, baseline IOP before filtering surgery, baseline number of glaucoma medications, VF mean deviation, and postoperative bleb procedures, including autologous bleb blood injection; use of sodium hyaluronate in the anterior chamber; suture lysis; needling; compression sutures; surgical revision; recovery time, defined as the time between the first autologous blood injection and hypotony resolution; and OCT scans of the macular region.

According to the World Glaucoma Association (WGA), we defined numerical hypotony as IOP < 6 mmHg for 2 consecutive examinations [20]. Hypotony maculopathy was defined by the appearance of choroidal folds in the macular region, assessed by the examination of the fundus oculi and OCT. Hypotony resolution was defined as the restoration of normal IOP and the absence of choroidal folds, as observed with OCT. An IOP spike was defined as an increase in IOP of ≥10.0 mmHg compared to baseline and/or an IOP value ≥28.0 mmHg [23]. Refractory to treatment was defined as the persistence of maculopathy.

### 2.1. Autologous Blood Injection Technique

**Figure 1:**
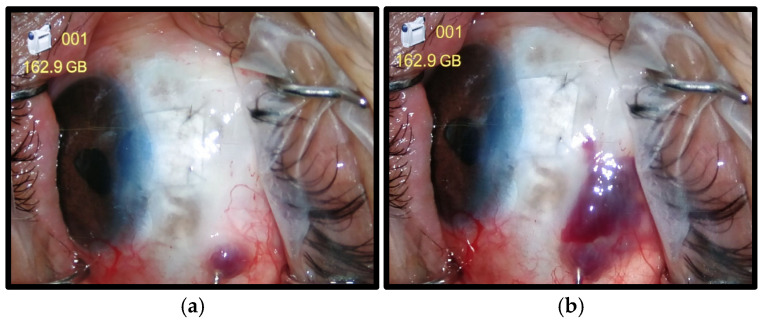

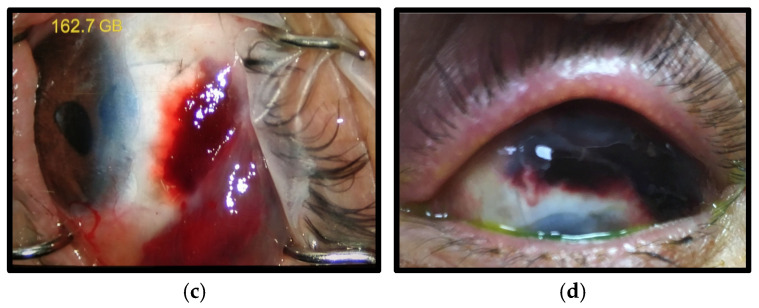
Autologous blood injection in the peribleb area: intra-operative procedure (**a**–**c**) and postoperative appearance (**d**).

After applying a tourniquet and disinfecting the skin of the patient’s arm, 3 mL of autologous blood was collected from the brachial vein. All trabeculectomy procedures were performed under topical anesthesia at the slit-lamp examination. The ocular skin and conjunctiva were disinfected with 5% iodopovidone (for 3 min). Approximately 0.5–1 mL of autologous blood was injected 5–8 mm away from the bleb via a 30-gauge needle to cover the entire bleb area, as shown in Figure 1. At the end of the procedure, a topical antibiotic was administered. The procedure was previously described [24]. 

### 2.2. Statistical Analysis

All data were analyzed using SPSS software, version 27.0 (IBM Corp., Armonk, NY, USA). Continuous variables were reported as mean ± SD and compared using the paired *t*-test, while categorical data were compared using χ^2^ with Fischer’s exact test. Snellen visual acuity measurements were converted to the logarithm of the minimum angle of resolution equivalents. A *p*-value ≤ 0.05 was considered statistically significant. The primary outcome of this study was the change in IOP following the surgical intervention for hypotony. Secondary outcomes included the resolution of maculopathy, improvement in best-corrected visual acuity (BCVA), and the need for further surgical or pharmacological interventions. These outcomes were assessed using clinical examination, OCT imaging, and IOP measurements over the follow-up period. Although this was a retrospective study, a post hoc power analysis was performed based on the observed change in IOP (primary outcome). A mean IOP reduction of 3 mmHg (standard deviation: 2.4 mmHg) was found. Using a two-sided paired *t*-test with a significance level of 0.05 and a power of 90%, the minimum sample size required to detect such a difference was calculated to be 9 patients. A similar post hoc power analysis was also performed for the resolution of maculopathy, which was present in all patients at baseline and persisted in only 22% of cases at follow-up. Based on this change in proportion, and assuming a significance level of 0.05 and a power of 90%, the minimum sample size required to detect such a reduction was calculated to be 5 patients.

## 3. Results

A total of nine patients (six male and three female) met the inclusion criteria and were included in our analysis (mean age 62.3 ± 17.0; 27–81 years). Most patients (*n* = 8) were Caucasian (*n* = 1 Hispanic). Glaucoma phenotypes included primary open-angle glaucoma (*n* = 6; POAG), primary angle-closure glaucoma (*n* = 2; PACG), and secondary glaucoma (*n* = 1; UG). Six patients underwent trabeculectomy, and three underwent phaco-trabeculectomy; all nine procedures were augmented with MMC (0.3 mg/mL, 3 min exposure). One patient had a previous stent gel implant. Five patients had myopia >6 diopters. The mean IOP was 24.37 ± 5.9 mmHg (range 20–35), despite maximum tolerated medical therapy (mean= 3.0 ± 0.7). See Table 1.

**Figure 2:**
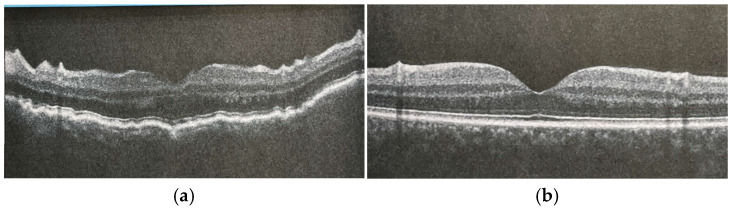
Pre-operative and postoperative macular OCT features showing hypotony maculopathy (**a**) and the resolution of the retinal and the choroidal folds (**b**).

At hypotony diagnosis, the mean IOP was considerably decreased (3.8 ± 1.5 mmHg; range 2–5), as shown in Figure 2. The mean baseline visual acuity was 0.9 ± 0.6 LogMAR (one patient presented with hand motion vision).

Autologous blood was injected once in seven patients, four times in patient #4, and five times in patient #1. No procedural complications were recorded.

Immediate significant increases in IOP were observed in eight patients (mean 8.3 ± 2.4 mmHg, *p* = 0.008); patient 1 maintained baseline IOP values. No IOP spikes were recorded. Treatment was refractory in two patients (patients 1 and 4) due to persistent maculopathy. Among the seven patients who experienced resolution of maculopathy, the mean visual acuity ranged from 0.1 to 0.3 (one patient had a full recovery of vision). The mean recovery time for maculopathy (defined as the disappearance of choroidal folds in the macular region on OCT) was 5.3 ± 1.4 weeks. Patients refractory to treatment underwent successful bleb surgical revision, with resolution of the maculopathy within 1 month, as shown in Table 2. 

## 4. Discussion

Autologous blood injection into an over-filtering bleb following trabeculectomy was found to be safe and effective in most patients (7/9 patients), without any procedural related complications or IOP spikes, in a homogeneous patient cohort, according to strict study criteria for patients with the same diagnosis, treated by the same surgeon, and following the same procedural protocol. Two cases of persistent maculopathy were observed in patients requiring multiple injections, and bleb surgical revisions were successfully performed.

Our study design defined strict inclusion and exclusion criteria for a homogeneous selection of patients with hypotony maculopathy due to over-filtering bleb. Further, compared to previous studies, we report OCT assessments for diagnosis and outcome, including the time to resolution of hypotony maculopathy. Several alternative treatments have been proposed for the management of clinically significant hypotony, including sutures [16,17,18,19], corneal patch grafts [20], the placement of nylon sutures to reduce flow [21], and complete surgical revisions [22]. Animal models have revealed that the peribleb injection of blood promotes cellular proliferation and collagen deposition [27,28,29,30]. In a rabbit model of post-trabeculectomy bleb leaks, the peribleb injection of autologous blood successfully sealed all leaks and was associated with increased fibroblast proliferation and collagen deposition around and within the bleb, suggesting a reparative effect potentially mediated by blood-derived trophic factors [29]. These results were further supported by a more recent experimental study, in which subconjunctival autologous blood injection led to increased conjunctival thickness and reduced transparency in a model of MMC-induced atrophic tissue. Over time, histological analysis showed a progressive increase in fibroblast density and collagen fiber deposition [30]. Collectively, these findings support the hypothesis that autologous blood may promote bleb healing through a reparative mechanism involving fibroblast recruitment and extracellular matrix remodeling. In addition, the volume of the blood itself creates mechanical compression over the flap, thus reducing the flow of the over-filtering bleb. As the timely resolution of hypotony maculopathy is crucial to avoid permanent vision loss, autologous blood injections were employed in the ocular surgery setting. Autologous blood injection is a quick and simple technique that can be performed in an office setting, does not require sutures, is a conjunctival-sparing approach with no tissue manipulation, and does not impact any further required surgical bleb revision in patients with persistent choroidal folds.

Following trabeculectomy, estimates from a 2-year prospective, randomized, multicenter, noninferiority study suggested that transient hypotony occurs in ~50% of patients treated with trabeculectomy [31]. Five-year treatment outcomes in the Tube Versus Trabeculectomy (TVT) study reported persistent hypotony in 12.4% of patients treated with trabeculectomy. Surgical revision for hypotony (with or without maculopathy) was reported to be necessary in ~7% of patients [31]. In the 5-year follow-up report of the Treatment of Advanced Glaucoma study published in 2024, a surgical bleb revision for hypotony was required in 6.6% of patients [32].

Retrospective studies have consistently reported lower incidences of hypotony compared to randomized controlled trials (RCTs), in the range of 1–10%. This is probably due to underestimated adverse events in retrospectively designed studies [33,34,35,36]. Further, it has been hypothesized that the risk of hypotony after trabeculectomy is elevated in myopic patients, as the thinner scleral wall of myopic eyes is at a higher risk of collapse at low IOP [37]. Five patients in our study had myopia.

In a review on the management of hypotony maculopathy, subconjunctival autologous blood injection was described as a conservative in-office technique for treating chronic hypotony following filtration surgery prior to considering more invasive surgical interventions [11].

Later reviews addressing the management of bleb-related complications and late-onset bleb leaks after trabeculectomy have also reported autologous blood injection as a potential approach for managing leaking or over-filtering blebs [38,39]. The first report of autologous blood injection for hypotony maculopathy due to over-filtering bleb was published in 1993. Treatment successfully increased IOP and improved visual acuity in four patients with chronic hypotony treated with MMC-augmented trabeculectomy [40]. One year later, the use of autologous blood injection was reported as a treatment for hypotony maculopathy with MMC-augmented trabeculectomy by Nuyts et al. [27]. Overall, 8 of the 22 patients included in the study received one injection, 14 received two injections, and 1 patient received three injections. In 68% of patients (*n* = 15), blood injection successfully resolved hypotony maculopathy; the remaining patients (31.8%; *n* = 7) were refractory to blood injection and were treated with surgical revision [27]. Choudhri et al. reported an unsatisfactory outcome of peribleb blood injection for the treatment of hypotony due to over-filtering or leaking blebs after glaucoma surgery, concluding that the procedure is of limited efficacy in resolving hypotony in patients with bleb leakage [28]. Soon after, Leen et al. reported partial successful hypotony resolution with autologous blood injection in 58% of patients (*n* = 7/12 patients) [41]. However, within 4 years, Okada et al. had confirmed peribleb blood injection to be an effective approach in resolving hypotony and preventing hypotony maculopathy in patients with hypotony due to over-filtering bleb with MMC-augmented trabeculectomy [42]. These confounding results may be partially explained by the inclusion of heterogeneous patient cohorts, including patients with hypotony due to excessive bleb filtration and bleb leakage, and small cohort sizes (Table 3).

In our case series, we did not record any procedure-related complications. Previous studies have observed infection, hyphemia, IOP spikes (up to 50 mmHg), and bleb perforation after bleb blood injection [27,42]. The authors hypothesize that the use of OVDs in the anterior chamber before the procedure increases the risk of IOP spikes. Although a mild intra-operative increase in IOP is not likely to be clinically relevant in most cases, the visual function of patients with advanced glaucoma may be compromised. We did not record any IOP spikes in our patient cohort. The authors suggest injecting small volumes of blood and avoiding OVDs in patients with advanced glaucoma. Surgical revision was necessary in two patients; of note, these patients were refractory to the first autologous blood injection, and despite multiple injection attempts, the patients continued to be refractory to treatment.

In contrast to previous studies, our patient cohort included patients treated by the same surgeon, undergoing the same augmented trabeculectomy treatment protocol, with hypotony maculopathy diagnosis, and treated with the same dose of MMC (0.3 mg/mL) for the same time period (3 min). Previous studies have reported lower [40,42] or higher [27] MMC dosages [28]. Alternatively, in some patient groups, only a portion of the cohort was treated with MMC augmentation [41]. Further, our cohort included patients with hypotony maculopathy due to over-filtering bleb only. Previous studies included patients with both over-filtering bleb and bleb leakage [27,41,42], and some did not include patients with maculopathy [42] or did not report the presence of hypotony maculopathy [40,41].

As guidelines for the management of hypotony due to over-filtering bleb are lacking, treatment choice is at the clinician’s discretion. Currently, there is substantial heterogeneity in reported treatment strategies, often employed in small cohort groups.

The main limitations of this study are its retrospective design and the small sample size. Although the number of included patients (*n* = 9) is in line with previous reports on similar clinical scenarios, the small sample may limit the generalizability of the findings and preclude more robust statistical analyses. Furthermore, the absence of a control group prevents a direct comparison with alternative treatment strategies for hypotony, restricting the ability to assess the relative efficacy of the proposed approach.

Additionally, the population included was highly selective, as all patients developed hypotony following MMC-augmented trabeculectomy. Therefore, the results may not be applicable to hypotony occurring after other types of filtering surgery or in the presence of different pathophysiological mechanisms.

Despite these limitations, the study offers valuable insight into a rare but clinically relevant condition and may serve as a useful foundation for future prospective controlled investigations. While these limitations should be acknowledged, they are partially offset by a number of methodological and clinical elements that support the value and applicability of the study.

Among these, the first lies in the focus on a clearly defined and clinically relevant population—patients with persistent hypotony following MMC-augmented trabeculectomy—addressing a challenging yet underreported complication. Second, outcomes were clearly pre-defined, with intraocular pressure (IOP) change identified as the primary endpoint and the resolution of maculopathy and visual acuity improvement as secondary outcomes. Third, a post hoc power analysis was performed based on the observed IOP change, confirming that the sample size (*n* = 9) was adequate to detect a clinically meaningful effect with 90% power. A similar analysis conducted for the resolution of maculopathy also supported the adequacy of the sample.

In addition, the follow-up period of 12 months provides insight into the medium-term efficacy and stability of the surgical outcomes. Of note, the diagnosis and follow-up of maculopathy were based on optical coherence tomography (OCT), offering an objective and reproducible evaluation that distinguishes this study from previous reports relying mostly on clinical examination. Furthermore, our findings support the potential clinical utility of autologous blood injection as a less invasive option in the management of hypotony prior to more extensive surgical revisions.

Finally, this study provides a detailed and practical description of the surgical approach, offering real-world guidance for clinicians managing similar cases.

Taken together, these elements contribute to the methodological clarity and clinical relevance of the study and provide novel insights into the structured management of a rare but impactful postoperative complication.

In this retrospective case series, we report the outcomes of autologous blood injection for the treatment of hypotony maculopathy following MMC-augmented trabeculectomy. Despite the small sample size, our findings suggest that this approach may significantly improve intraocular pressure and lead to maculopathy resolution in selected patients. These results support the potential role of autologous blood injection as a therapeutic option prior to more invasive surgical revisions. Further prospective studies are needed to confirm these findings and refine treatment indications.

## Figures and Tables

**Table 1:** Baseline patient characteristics prior to trabeculectomy.

Patient (*n*)	Age (yrs)	Sex (M, F)	Ethnicity	Glaucoma Phenotype	Myopia >6D	IOP at Baseline for Trab (mmHg)	IOP-Lowering Drugs (n)	Previous Surgery	Principal Surgery	MMC Dose and Exposure Time
**1**	63	F	C	POAG	Yes	20	3	Phaco, SLT	Trab	0.3 mg/mL, 3 min
**2**	78	M	C	PACG	No	34	2	iridotomy, phaco, stent gel implant	Trab	0.3 mg/mL, 3 min
**3**	71	F	C	POAG	Yes	22	4	Phaco	Trab	0.3 mg/mL, 3 min
**4**	48	M	C	POAG	Yes	20	2	/	Phaco-trab	0.3 mg/mL, 3 min
**5**	27	M	C	UG	No	35	3	/	Trab	0.3 mg/mL, 3 min
**6**	56	M	H	POAG	No	21	3	/	Phaco-trab	0.3 mg/mL, 3 min
**7**	63	M	C	POAG	No	26	4	Phaco	Trab	0.3 mg/mL, 3 min
**8**	74	F	C	PACG	Yes	20	3	Iridotomy	Phaco-trab	0.3 mg/mL, 3 min
**9**	81	M	C	POAG	Yes	24	3	Phaco	Trab	0.3 mg/mL, 3 min
***Mean*** ***± SD***	*62.3 ± 17.0*					*24.7 ± 5.9*	*3.0 ± 0.7*			

M: male, F: female; C: Caucasian; H: Hispanic; MMC: mitomycin C; POAG: primary open-angle glaucoma; PACG: primary angle-closure glaucoma; UG: uvetic glaucoma; Phaco: phacoemulsification; Trab: trabeculectomy; SLT: selective laser trabeculoplasty; IV: intravitreal; *n*: number.

**Table 2:** Pre-, post-, and peri-procedural clinical data for patients with hypotony maculopathy previously treated with trabeculectomy.

Patients		At Maculopathy Diagnosis (Pre-Procedure)			Autologous Blood Injection (Procedure)			Outcomes (Post-Procedure)			Outcomes (Follow-Up)	
	IOP		Visus (LogMAR)	AC Refill with OVD (*n*)		Blood Injection (n)	IOP		Visus (LogMAR)	Persistent Maculopathy		Time to Maculopathy Resolution (Weeks)
**1**	5		1.0	1		5	5		0.7	Yes		Unresolved
**2**	3		1.0	3		1	8		0.1	No		4
**3**	2		2.3	0		1	10		0.4	No		5
**4**	3		0.7	3		4	7		0.4	Yes		Unresolved
**5**	5		0.3	1		1	8		0.0	No		6
**6**	5		0.4	0		1	8		0.2	No		8
**7**	3		0.9	2		1	6		0.3	No		4
**8**	6		0.7	1		1	13		0.1	No		5
**9**	2		1.0	2		1	10		0.2	No		5
***Mean***	*3.8 ± 1.5*		*0.9 ± 0.6*				8.3 ± 0.4 *		*0.3 ± 0.2 #*			*5.3 ± 1.4*
***Percentage***										*22.2% °*		

AC: anterior chamber; OVD: ophthalmic viscoelastic device; *n*: number; * IOP pre vs. post, *p =* 0.008 (paired *t*-test); # visus pre vs. post, *p* = 0.14 (paired *t*-test); ° maculopathy pre vs. post, *p* = 0.06 (Fisher’s exact test).

**Table 3:** Selected studies in the literature reporting autologous blood injection for the management of hypotony after trabeculectomy.

Study (Author, Publication Year)	Definition of Hypotony	MMC (n Patients), Dose, and Exposure Time	Presence of Bleb Leakage (n Eyes)	Presence of Hypotony Maculopathy	Use of OCT
**Wise, 1993 [40]**	Not reported	4/4 0.25 mg/mL For 5 min	Not reported	Not reported	No
**Nuyts, 1994 [27]**	≤6 mmHg	34/34 0.5 mg/mL For 5 min	13/34	Yes	No
**Leen, 1995 [41]**	Not reported	5/12 Not reported Not reported	7/12	Not reported	No
**Choudhri, 1997 [28]**	Not reported	5/10 Not reported Not reported	3/10	Yes	No
**Okada, 2001 [42]**	Not reported	5/5 0.2 mg/mL For 5 min	No	No	No
***Present study***	*<6 mmHg*	*9/9* *0.4 mg/mL* *For 3 min*	*No*	*Yes*	*Yes*

MMC: mitomycin C; OCT: optical coherence tomography.

## Data Availability

The data presented in this study are available on request from the corresponding author. The data are not publicly available due to privacy restrictions.

## References

[1] Tham Y.-C., Li X., Wong T.Y., Quigley H.A., Aung T., Cheng C.-Y. (2014). Global Prevalence of Glaucoma and Projections of Glaucoma Burden through 2040. Ophthalmology.

[2] Spaeth G.L. (2021). European Glaucoma Society Terminology and Guidelines for Glaucoma, 5th Edition. Br. J. Ophthalmol..

[3] Sacchi M., Agnifili L., Brescia L., Oddone F., Villani E., Nucci P., Mastropasqua L. (2020). Structural imaging of conjunctival filtering blebs in XEN gel implantation and trabeculectomy: A confocal and anterior segment optical coherence tomography study. Graefes Arch. Clin. Exp. Ophthalmol..

[4] Jampel H.D., Musch D.C., Gillespie B.W., Lichter P.R., Wright M.M., Guire K.E. (2005). Perioperative Complications of Trabeculectomy in the Collaborative Initial Glaucoma Treatment Study (CIGTS). Am. J. Ophthalmol..

[5] Gedde S.J., Herndon L.W., Brandt J.D., Budenz D.L., Feuer W.J., Schiffman J.C. (2012). Postoperative Complications in the Tube Versus Trabeculectomy (TVT) Study During Five Years of Follow-up. Am. J. Ophthalmol..

[6] Chen D.Z., Sng C.C.A. (2017). Safety and Efficacy of Microinvasive Glaucoma Surgery. J. Ophthalmol..

[7] Aref A.A., Gedde S.J., Budenz D.L. (2017). Glaucoma Drainage Implant Surgery. Dev. Ophthalmol..

[8] Palma A., Covello G., Posarelli C., Maglionico M.N., Agnifili L., Figus M. (2024). Is the Advent of New Surgical Procedures Changing the Baseline Features of Patients Undergoing First-Time Glaucoma Surgery?. J. Clin. Med..

[9] Okonkwo O.N., Tripathy K. (2024). Ocular Hypotony. StatPearls.

[10] Thomas M., Vajaranant T.S., Aref A.A. (2015). Hypotony Maculopathy: Clinical Presentation and Therapeutic Methods. Ophthalmol. Ther..

[11] Costa V.P., Arcieri E.S. (2007). Hypotony maculopathy. Acta Ophthalmol. Scand..

[12] Tosi G.M., Schiff W., Barile G., Yoshida N., Chang S. (2005). Management of Severe Hypotony with Intravitreal Injection of Viscoelastic. Am. J. Ophthalmol..

[13] Küçükerdönmez C., Beutel J., Bartz-Schmidt K.U., Gelisken F. (2009). Treatment of chronic ocular hypotony with intraocular application of sodium hyaluronate. Br. J. Ophthalmol..

[14] Elving-Kokke K., Sas-Meertens M., de Beer F., van Rijn L., de Boer J., Visser E.-S. (2019). The treatment of ocular hypotony after trabeculectomy with a scleral lens: A case series. Contact Lens Anterior Eye.

[15] Gollakota S., Garudadri C.S., Mohamed A., Senthil S. (2017). Intermediate Term Outcomes of Early Posttrabeculectomy Bleb Leaks Managed by Large Diameter Soft Bandage Contact Lens. J. Glaucoma.

[16] Maheshwari D., Shyam P., Pawar N., Ramakrishnan R. (2022). Transconjunctival flap sutures: A novel technique to combat hypotony. Indian J. Ophthalmol..

[17] Eha J., Hoffmann E.M., Wahl J., Pfeiffer N. (2008). Flap suture—A simple technique for the revision of hypotony maculopathy following trabeculectomy with mitomycin C. Graefes Arch. Clin. Exp. Ophthalmol..

[18] Yu J.T., Mercieca K., Au L. (2018). Conjunctival bleb compression sutures: An effective method of addressing hypotony after trabeculectomy or trabeculectomy-related procedures. Eur. J. Ophthalmol..

[19] Yu J.T.S., Au L. (2020). Conjunctival bleb compression as a treatment for hypotony post XEN45 implant in uveitic glaucoma. Eur. J. Ophthalmol..

[20] Bochmann F., Kaufmann C., Kipfer A., Thiel M.A. (2014). Corneal Patch Graft for the Repair of Late-onset Hypotony or Filtering Bleb Leak After Trabeculectomy: A New Surgical Technique. J. Glaucoma.

[21] Iyer J.V., Pitha I., Jampel H.D., Boland M.V. (2019). Management of Tube-Related Hypotony Using Ab Interno Placement of Multifilament Nylon Suture to Reduce Flow. Ophthalmol. Glaucoma.

[22] Cohen J.S., Shaffer R.N., Hetherington J., Hoskins D. (1977). Revision of Filtration Surgery. Arch. Ophthalmol..

[23] Suleman A., Aluyi-Osa G., Ashipa F., Spadea L., Gagliano C., D’esposito F., Zeppieri M., Musa M. (2024). Autologous blood in the management of ocular surface disorders. World J. Exp. Med..

[24] Abdalrahman O., E Rodriguez A., Del Barrio J.L.A., Alio J.L. (2018). Treatment of chronic and extreme ocular hypotension following glaucoma surgery with intraocular platelet-rich plasma: A case report. Eur. J. Ophthalmol..

[25] Martinez de la Casa J.M., Feijoó J.G., Gómez A.C., Benítez J.M., Valdizán C.M., Sánchez J.G. (2003). Hypotony maculopathy diagnosed by optical coherence tomography. Arch. Soc. Esp. Oftalmol..

[26] Weyll M., Gilio A., Barbosa A., Nicoli A.A., Silveira R.C. (2006). Detection of subclinical hypotony maculopathy with OCT III after filtration surgery. Arq. Bras. Oftalmol..

[27] Nuyts R.M.M.A., Greve E.L., Geijssen H.C., Langerhorst C.T. (1994). Treatment of Hypotonous Maculopathy After Trabeculectomy With Mitomycin C. Am. J. Ophthalmol..

[28] Choudhri S.A., Herndon L.W., Damji K.F., Allingham R.R., Shields M.B. (1997). Efficacy of Autologous Blood Injection for Treating Overfiltering or Leaking Blebs After Glaucoma Surgery. Am. J. Ophthalmol..

[29] Doyle J.W., Smith M.F., Garcia J.A., Sherwood M.B., Lau T. (1996). Injection of autologous blood for bleb leaks in New Zealand white rabbits. Invest. Ophthalmol. Vis. Sci..

[30] Hwang J.-M., Kee C. (2006). Injection of cultured autologous fibroblasts into the subconjunctival space of rabbits treated with mitomycin C. Am. J. Ophthalmol..

[31] Baker N.D., Barnebey H.S., Moster M.R., Stiles M.C., Vold S.D., Khatana A.K., Flowers B.E., Grover D.S., Strouthidis N.G., Panarelli J.F. (2021). Ab-Externo MicroShunt versus Trabeculectomy in Primary Open-Angle Glaucoma. Ophthalmology.

[32] King A.J., Hudson J., Azuara-Blanco A., Burr J., Kernohan A., Homer T., Shabaninejad H., Sparrow J.M., Garway-Heath D., Barton K. (2024). Evaluating Primary Treatment for People with Advanced Glaucoma. Ophthalmology.

[33] Kirwan J.F., Lockwood A.J., Shah P., Macleod A., Broadway D.C., King A.J. (2013). Trabeculectomy in the 21st Century. Ophthalmology.

[34] Fontana H., Nouri-Mahdavi K., Caprioli J. (2006). Trabeculectomy With Mitomycin C in Pseudophakic Patients With Open-angle Glaucoma: Outcomes and Risk Factors For Failure. Am. J. Ophthalmol..

[35] Sacchi M., Monsellato G., Villani E., Lizzio R.A.U., Cremonesi E., Luccarelli S., Nucci P. (2022). Intraocular pressure control after combined phacotrabeculectomy versus trabeculectomy alone. Eur. J. Ophthalmol..

[36] Wanichwecharungruang B., Ratprasatporn N. (2021). 24-month outcomes of XEN45 gel implant versus trabeculectomy in primary glaucoma. PLoS ONE.

[37] Sacchi M., Fea A.M., Monsellato G., Tagliabue E., Villani E., Ranno S., Nucci P. (2023). Safety and Efficacy of Ab Interno XEN 45 Gel Stent in Patients with Glaucoma and High Myopia. J. Clin. Med..

[38] Bochmann F., Azuara-Blanco A. (2012). Interventions for late trabeculectomy bleb leak. Cochrane Database Syst. Rev..

[39] Leung D.Y., Tham C.C. (2013). Management of bleb complications after trabeculectomy. Semin. Ophthalmol..

[40] Wise J.B. (1993). Treatment of Chronic Postfiltration Hypotony by Intrableb Injection of Autologous Blood. Arch. Ophthalmol..

[41] Leen M.M. (1995). Management of Overfiltering and Leaking Blebs With Autologous Blood Injection. Arch. Ophthalmol..

[42] Okada K., Tsukamoto H., Masumoto M., Jian K., Okada M., Mochizuki H., Mishima H.K. (2001). Autologous blood injection for marked overfiltration early after trabeculectomy with mitomycin C. Acta Ophthalmol. Scand..

